# *In Vitro* Screening for Compounds That Enhance Human L1 Mobilization

**DOI:** 10.1371/journal.pone.0074629

**Published:** 2013-09-11

**Authors:** Natsuko Terasaki, John L. Goodier, Ling E. Cheung, Yue J. Wang, Masaki Kajikawa, Haig H. Kazazian, Norihiro Okada

**Affiliations:** 1 Department of Biological Science, Graduate School of Bioscience and Biotechnology, Tokyo Institute of Technology, Yokohama, Kanagawa, Japan; 2 McKusick-Nathans Institute for Genetic Medicine, Johns Hopkins University School of Medicine, Baltimore, Maryland, United States of America; 3 Department of Life Sciences, National Cheng Kung University, Tainan, Taiwan; Louisiana State University, United States of America

## Abstract

The Long interspersed element 1 (LINE1 or L1) retrotransposon constitutes 17% of the human genome. There are currently 80–100 human L1 elements that are thought to be active in any diploid human genome. These elements can mobilize into new locations of the genome, resulting in changes in genomic information. Active L1s are thus considered to be a type of endogenous mutagen, and L1 insertions can cause disease. Certain stresses, such as gamma radiation, oxidative stress, and treatment with some agents, can induce transcription and/or mobilization of retrotransposons. In this study, we used a reporter gene assay in HepG2 cells to screen compounds for the potential to enhance the transcription of human L1. We assessed 95 compounds including genotoxic agents, substances that induce cellular stress, and commercially available drugs. Treatment with 15 compounds increased the L1 promoter activity by >1.5-fold (*p*<0.05) after 6 or 24 hours of treatment. In particular, genotoxic agents (benzo[a]pyrene, camptothecin, cytochalasin D, merbarone, and vinblastine), PPARα agonists (bezafibrate and fenofibrate), and non-steroidal anti-inflammatory drugs (diflunisal, flufenamic acid, salicylamide, and sulindac) induced L1 promoter activity. To examine their effects on L1 retrotransposition, we developed a high-throughput real-time retrotransposition assay using a novel secreted Gaussia luciferase reporter cassette. Three compounds (etomoxir, WY-14643, and salicylamide) produced a significant enhancement in L1 retrotransposition. This is the first study to report the effects of a wide variety of compounds on L1 transcription and retrotransposition. These results suggest that certain chemical- and drug-induced stresses might have the potential to cause genomic mutations by inducing L1 mobilization. Thus, the risk of induced L1 transcription and retrotransposition should be considered during drug safety evaluation and environmental risk assessments of chemicals.

## Introduction

Long interspersed element 1 (LINE1 or L1) is a non-long terminal repeat retrotransposon. L1 constitutes 17% of the human genome [1], and 80–100 human L1 elements are estimated to be potentially active in the average human [2], [3]. L1s possess a 5′ untranslated region (UTR), two open reading frames (ORF1 and ORF2), and a 3′ UTR with poly(A) signal and tail [4], [5]. The 5′ UTR has an internal promoter, and ORF2 encodes endonuclease [6] and reverse transcriptase [7] activities, both of which are required for autonomous retrotransposition [8]. L1s are likely to be integrated by a mechanism termed target-primed reverse transcription (TPRT) [9], [10]. During TPRT, L1 endonuclease makes a nick in the first strand of the target site in the genomic DNA, and the 3′ hydroxyl at the nick is used as a primer for reverse transcription by L1-encoded reverse transcriptase. Then, second-strand cleavage occurs and the cDNA is integrated by a largely unknown mechanism. After second-strand DNA synthesis, retrotransposition is completed by the DNA repair system of the host [11]. Active L1 elements are thus considered a type of endogenous mutagen. Indeed, natural L1 insertions into genes have been discovered in many disease cases [12]–[14] which were caused by retrotransposition that likely occurred in the germ line. For example, progeny of active L1s have been found inserted into the genes encoding factor VIII causing hemophilia A [15], dystrophin causing muscular dystrophy [16], and pyruvate dehydrogenase X causing pyruvate dehydrogenase complex deficiency [17]. Several lines of evidence suggest that retrotransposition can occur in some somatic cells, including neuronal progenitor cells and certain cancers [18]–[22]. Somatic L1 insertions that occur in tumor suppressor genes may be causal for certain tumors or hasten the progression of tumorigenesis [21], [23].

L1 transcription and/or mobilization is upregulated by certain types of stress: for example, genotoxic stress, which is induced by benzo[a]pyrene, UV light [24], [25], gamma radiation [26], and X-ray irradiation [27]; oxidative stress, which is induced by H_2_O_2_ [28], heavy metals [29], 6-formylindolo[3,2-b]carbazole [30], and in the mouse, exercise stress [31]. Physiological stresses such as heat shock [32] can induce retrotransposons other than L1 in various species [33]. In addition, L1 ORF1 protein is localized in cytoplasmic stress granules, which often form under stress conditions [34].

Chemical- or drug-induced retrotransposition of L1 into a functional gene in germ cells or somatic cells might cause teratogenicity or disease, including cancer. However, it is still unclear what kinds of compounds affect L1 retrotransposition, and the risk of inducing retrotransposition by compounds has not been considered during drug or chemical safety assessment.

In this study, we used a reporter gene assay in HepG2 cells to investigate compounds for their potential to enhance the transcription of human L1. We examined 95 compounds, each of which was categorized as a genotoxic agent, cellular stress agent, or drug. Of these compounds, 54 are now available commercially. Among the 95 compounds, 15 compounds (8 of which are commercially available drugs) increased the L1 promoter activity. To examine their effect on L1 retrotransposition, we designed a retrotransposition assay using a novel secreted Gaussia luciferase reporter cassette. Importantly, we found that salicylamide not only increased L1 promoter activity but also slightly enhanced L1 retrotransposition in HeLa cells. Because this drug is widely used as an anti-pyretic analgesic, the data presented here suggest a new paradigm for drug assessment.

## Materials and Methods

### Plasmid Constructs

pGL4.11-L1.3 5′ UTR contains the 5′ UTR of L1.3 upstream of a Firefly luciferase (FLuc) gene. The 5′ UTR was amplified by PCR using a forward primer (5′-ATGCCTCGAGGGGGGAGGAGCCAAGATGGCCG-3′) that contains the *Xho*I restriction site and a reverse primer (5′-GCATAAGCTTCTTTGTGGTTTTATCTACTTTTGGTCTTTG-3′) that contains the *Hin*dIII restriction site. The PCR product was cloned into *Xho*I- and *Hin*dIII-digested linearized pGL4.11 (Promega). pCEP4/L1.3mneoI_400_/ColE1 [35] was used as the template for the PCR. pGL4.74 (Promega) containing the herpes simplex virus thymidine kinase (HSV-TK) promoter and the Renilla luciferase (RLuc) gene was used as an internal control vector for the luciferase reporter gene assay.

99-PUR-RPS-*mGLucI* contains L1_RP_ [36] with a Gaussia luciferase (GLuc) retrotransposition cassette in the 99-PUR backbone, which has a puromycin resistance gene. To construct the *mGLucI* retrotransposition cassette, the SV40 poly(A) signal of the construct pORF5-Fcy::Fur (InvivoGen) was replaced with a thymidine kinase (TK) poly(A) signal. Next, an *Eco*ICRI restriction site was introduced into the GLuc gene [37] in the construct pGLuc-Basic (New England BioLabs) by QuickChange (Stratagene) mutagenesis (this introduced a D-to-E mutation at residue 39 of GLuc). The modified GLuc gene was extracted from pGLuc-Basic by PCR and swapped for the Fcy::Fur gene (between flanking *Nco*I and *Nhe*I sites) of pORF5-Fcy::Fur, immediately downstream of the EF-1α/eIFg hybrid promoter. The chimeric mini-intron of the plasmid psiCHECK-2 (Promega) was isolated and cloned in the *Eco*ICRI site of GLuc in the antisense orientation. The entire reporter cassette was then extracted with flanking *Pac*I and *Asi*SI restriction enzyme sites, blunted, and cloned in a *Bst*Z17I site in the 3′ UTR of the active L1_RP_ contained within the vector 99-PUR [36], [38].

99-PUR-JM111-*mGLucI* is the JM111 mutant of 99-PUR-RPS-*mGLucI*. The JM111 mutant contains two missense mutations in L1_RP_ ORF1 that abolish retrotransposition [8]. pCMV-GLuc (New England BioLabs) constitutively expresses Gaussia luciferase under a CMV promoter.

pSV40-CLuc (New England BioLabs), which contains an SV40 promoter and Cypridina luciferase (CLuc) [39], was used as an internal control vector for the high-throughput 96-well retrotransposition assay.

Ultimate ORF cDNA clones (Invitrogen) were V5-tagged on their N-termini by shuttling them from pENTR221 vector into pcDNA3.1/nV5-DEST using Gateway Technology (Invitrogen) as previously described [40]. Different forms of this construct expressed the following genes: DDX39A (Invitrogen Ultimate ORF cDNA Clone number IOH3477), HNRNPK (IOH3427), HNRNPU (IOH3430), ILF2 (1OH3433), and MOV10 (IOH4005). Plasmids were purified with a Plasmid Maxi kit (Qiagen).

### Cell Culture

Human hepatocellular carcinoma HepG2 cells (ATCC) were grown in minimum essential medium supplemented with 1 mM sodium pyruvate, 0.1 mM non-essential amino acids, 2 mM l-glutamine, and 10% fetal bovine serum (Life Technologies, Inc.). Human adenocarcinoma HeLa cells (ATCC) and HEK293T cells (ATCC) were grown in Dulbecco’s modified Eagle’s medium with 10% fetal bovine serum (Life Technologies, Inc.). Cells were grown at 37°C in a humidified 5% CO_2_ atmosphere. Cells were grown in a T75 or T225 flask to ∼80% confluence and passaged using a 0.25% trypsin/0.02% EDTA solution (Sigma-Aldrich).

### Test Compounds

Most of the compounds were purchased from Sigma-Aldrich, Wako Pure Chemical Industries, Ltd., or EMD Millipore. All test compounds were solubilized in dimethyl sulfoxide (DMSO) and then stored at −20°C until use. They were diluted with DMSO to different concentrations and added to the culture medium at a 1/1000 (vol/vol) dilution just before use.

### Luciferase Reporter Gene Assay

HepG2 (0.33×10^4^ cells/100 µl/well) or HeLa (0.5×10^4^ cells/100 µl/well) cells were seeded on 96-well plates. After a 24-hour incubation, cells were cotransfected with 30 ng pGL4.11-L1.3 5′ UTR and 30 ng pGL4.74 with 0.18 µl FuGENE6 reagent (Roche) per well. At 24 hours post-transfection, cells were exposed to the various concentrations of test compounds. After 6 or 24 hours of treatment, FLuc and RLuc luminescence were measured by Multilabel Counter 1420 ARVO (PerkinElmer) using the Dual-Glo Luciferase Assay System (Promega).

Assays were performed in duplicate or quadruplicate and repeated at least twice. To analyze the effects of compounds on L1 promoter activity, the ratio of FLuc luminescence to RLuc luminescence, which was used as an internal control, was calculated, and this ratio was normalized to the ratio of the vehicle (DMSO) control. If the addition of the compound reduced the RLuc activity to <50% of the DMSO control, the data at those concentrations were not used for analysis.

### Retrotransposition Assay

Initially, Gaussia luciferase L1 reporter constructs were tested for efficacy in HEK293T cells and 6-well plates. One µg of 99-PUR-RPS*-mGLucI* or 99-PUR- JM111*-mGLucI* was cotransfected with 0.5 µg of empty vector (pcDNA3) or V5-tagged Ultimate ORF cDNA construct (four replicate wells each). At 24 hours post-transfection, 3 mls of media were replaced, and at subsequent time-points 50 µl of media was sampled from each well for time-course determination of luminescence. All readings for a single experiment were made at the same time using the BioLux Gaussia luciferase assay kit (New England BioLabs). Readings were adjusted for background luminescence of media alone.

To screen chemical compounds for effects on retrotransposition, HeLa cells (0.5×10^4^ cells/100 µl/well) were seeded on a 96-well plate. After a 24-hour incubation, cells were cotransfected with 100 ng 99-PUR-RPS-*mGLucI* or 99-PUR-JM111-*mGLucI* and 2 ng pSV40-CLuc with 0.3 µl FuGENE6 reagent per well. At 24 hours post-transfection, the medium was replaced with fresh medium containing 1 µg/ml puromycin and various concentrations of test compounds. Untreated cells were included in each plate for normalization between plates and experiments. After a 2-day culture, cells were exposed to fresh medium containing only the test compounds. After an additional 3-day incubation, two 20-µl aliquots of the medium were sampled to measure GLuc and CLuc. Luminescence was measured with the Multilabel Counter 1420 ARVO using the BioLux Gaussia luciferase assay kit (New England BioLabs) and BioLux Cypridina luciferase assay kit (New England BioLabs).

Four or six independent assays were performed in quadruplicate. To evaluate the effects of compounds on L1 retrotransposition activity, the ratio of GLuc luminescence to the internal control CLuc luminescence was calculated. Then the ratio was normalized based on the luminescence of untreated cells. The percentage of the normalized value for each treatment relative to the vehicle (DMSO) control was calculated. After luminescence in the medium was measured for the L1 retrotransposition assay, cell viability was determined (see below). The concentration at which cell viability was >80% was used to evaluate L1 retrotransposition activity.

### PCR and Cell Immunofluorescence

To confirm removal of the mini-intron, and therefore genomic insertion of *mGLucI*-tagged L1s, HeLa cells were harvested at four days post-transfection and their DNA were extracted with a DNeasy Tissue Kit (Qiagen). PCR was performed using primers that flank the intron: sc-F4 (CTTTCCGGGCATTGGCTTCC) and sc-R4 (CAAGCCCACCGAGAACAACG). Digesting the genomic DNA prior to PCR with restriction enzymes that cut within the intron (*Ban*I or *Bsp*T107I) increased the amount of 149-base pair (bp) spliced product and reduced the amount of 282-bp unspliced product.

Immunofluorescence techniques have been described [34]. Primary and secondary antibodies were α-GLuc (New England BioLabs) and DyLight 488-conjugated α-rabbit IgG (Jackson ImmunoResearch Laboratories), respectively.

### Cell Viability

Cell viability was determined by measuring the amount of ATP present using the Cell Titer-Glo Luminescent Cell Viability Assay (Promega). Luminescence was measured with the Multilabel Counter 1420 ARVO. The percentage of luminescence in treated wells relative to that in control wells was used to determine cell viability. Cell viability was evaluated for four or six independent experiments.

### Statistical Analysis

Statistical analyses were performed with Student’s *t*-test between the control and treated wells. When two or more concentrations were used, Dunnett’s multiple comparison test was applied. Statistical analyses were performed with SAS 9.1.3 software (SAS Institute Inc.), with *p*-values <0.05 considered significant.

## Results

### Effect of Genotoxic Agents on L1 Promoter Activity

We wished to determine the effects of a wide selection of environmental, genotoxic, and therapeutic compounds, including many of common human exposure, on the activity of the LINE1 retrotransposon, an insertional mutagen linked to the etiology of human disease and possibly cancers. To begin, we cloned the 5′ UTR of L1.3, one of the most active human L1s [41], upstream of a Firefly luciferase gene (FLuc). After 6 or 24 hours from the transfection of HepG2 cells with this vector, L1 promoter activity was clearly detected by FLuc expression (Fig. S1). RLuc luminescence co-expressed from vector pGL4.74 was used to normalize the transfection efficiency and to adjust for the number of cells in different wells. We used 96-well plates for high-throughput assessment.

**Figure 1:**
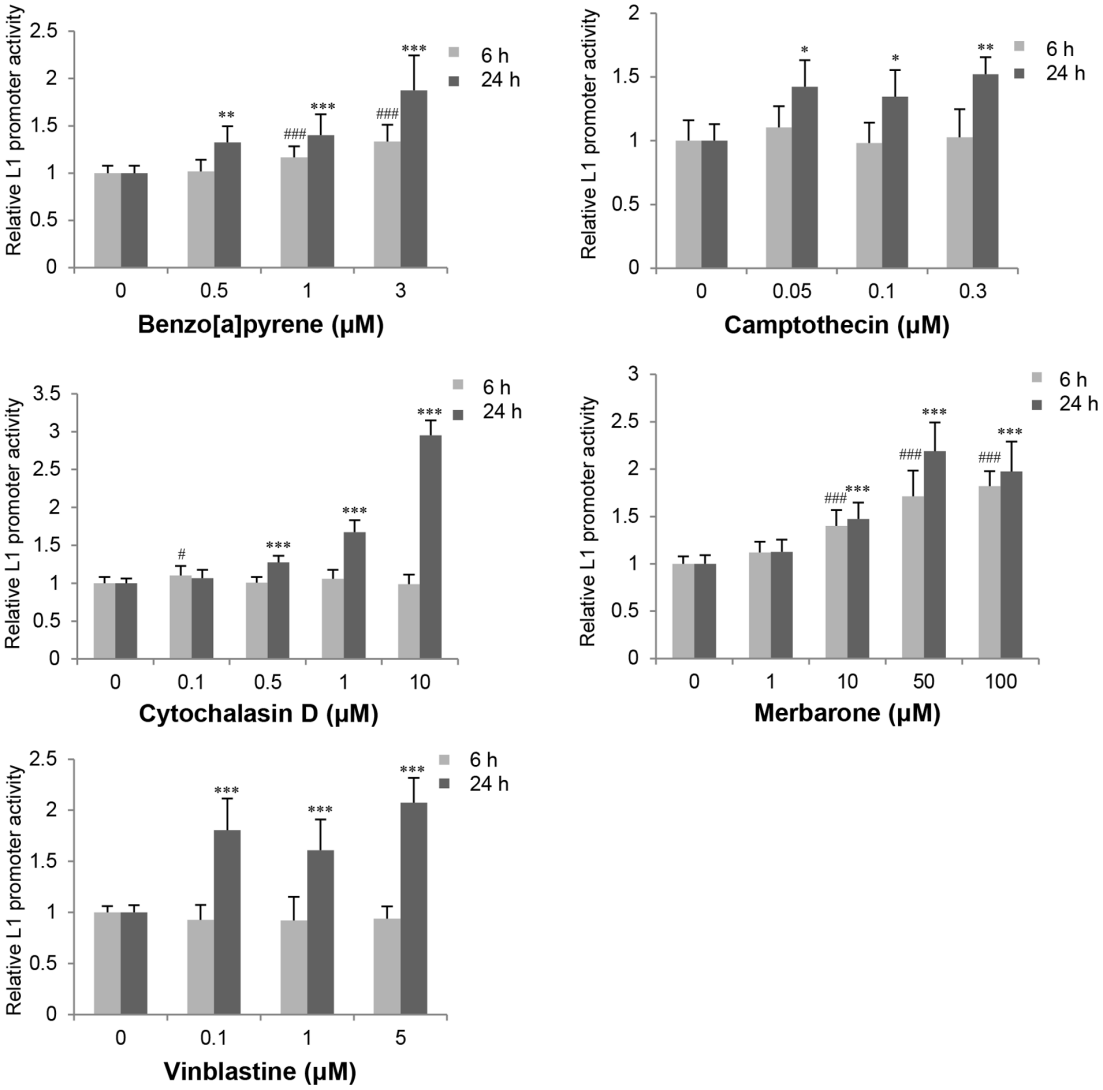
Effects of genotoxic agents on L1 promoter activity in HepG2 cells. L1 promoter activity 6 and 24 hours after treatment was evaluated by the luciferase reporter gene assay. Data are the average ratio ± the standard deviation (SD) of the L1 promoter activity in the presence of the test compound relative to that in the presence of the vehicle control from four independent experiments. Each experiment was carried out in duplicate (n = 8), except in the case of 0.5 µM benzo[a]pyrene and camptothecin and 0.1 and 5 µM vinblastine (at least two independent experiments in duplicate, n = 4 or more). Statistically significant differences as compared with the vehicle control for 6-hour and 24-hour treatments are indicated by *and #, respectively (*or #, *p*<0.05; **p<0.01; ***or ###, *p*<0.001).

Using the system described above, we screened 95 compounds for effects on L1 promoter activity. Among these 95 compounds, 21 genotoxic agents with various mechanisms of action, such as DNA alkylation, DNA crosslinking, and DNA topoisomerase inhibition, were evaluated (Table 1). Five genotoxic agents (benzo[a]pyrene, camptothecin, cytochalasin D, merbarone, and vinblastine) increased L1 promoter activity by >1.5-fold (*p*<0.05) after exposure for 6 hours and/or 24 hours (Fig. 1). Among 9 compounds which induced >1.2-fold increase (*p*<0.05) in L1 promoter activity listed in Table 1, actinomycin D, cisplatin, mitomycin C, and vinblastine are used clinically as anticancer drugs.

**Table 1:** Effects of various genotoxic agents on L1 promoter activity.

Mechanism of action	Compound name	L1 promoter activity	
		6 h	24 h
DNA alkylator	Busulfan	N.S.	N.S.
	Cyclophosphamide	N.S.	−
	Methyl methanesulfonate	N.S.	N.S.
DNA crosslinker	Mitomycin C	N.S.	+
DNA binder	Actinomycin D	N.S.	+
	Cisplatin	+	N.S.
	Doxorubicin	N.S.	− −
DNA antimetabolite	Cytosine arabinoside	N.S.	N.S.
	Fluorouracil	N.S.	−
	Methotrexate	N.S.	N.S.
	6-Thioguanine	+	N.S.
DNA topoisomerase I inhibitor	Camptothecin	N.S.	+ +
DNA topoisomerase II inhibitor	Etoposide	N.S.	N.S.
	Merbarone	+ +	+ +
DNA intercalator	Benzo[a]pyrene	+	+ +
Reverse transcriptase inhibitor	Azidothymidine	N.S.	N.S.
Nucleic acid analog	Ganciclovir	N.S.	N.S.
Microtubule assembly inhibitor	Colchicine	N.S.	N.S.
	Taxol	N.S.	−
	Vinblastine	N.S.	+ +
Actin polymerization inhibitor	Cytochalasin D	N.S.	+ +

The effects of treatment for 6 and 24 hours on L1 promoter activity relative to the vehicle control were evaluated with the luciferase reporter gene assay.+and++, 1.2- to 1.5-fold increase and >1.5-fold increase at one or more concentrations, respectively (*p*<0.05); − and − −, 1.2- to 1.5-fold decrease and >1.5-fold decrease at one or more concentrations, respectively (*p*<0.05); N.S., no significant change. The data for the L1 reporter gene assay at all test concentrations are shown in Dataset S1.

Benzo[a]pyrene, a DNA intercalator, increased the L1 promoter activity in a dose-dependent manner by >1.5-fold at 24 hours. This is consistent with a previous report, which showed that Benzo[a]pyrene activates mouse L1 transcription [24]. Camptothecin, a DNA topoisomerase I inhibitor, enhanced L1 promoter activity >1.5-fold only after 24 hours of treatment. Merbarone, a DNA topoisomerase II inhibitor, enhanced L1 promoter activity in a dose-dependent manner to 50 µM, and ∼2-fold increases were observed at 6 and 24 hours of treatment. In contrast, etoposide, which is also a DNA topoisomerase II inhibitor, did not affect L1 promoter activity in this assay, although this compound has been reported to increase Alu retrotransposition [42]. Cytochalasin D, an actin polymerization inhibitor, and vinblastine, a microtubule assembly inhibitor, both of which inhibit reconstruction of the cytoskeleton, at their highest concentrations enhanced L1 promoter activity by 3-fold and 2-fold, respectively, after 24 hours of treatment (Fig. 1). The data for all 95 compounds including genotoxic agents are shown in Dataset S1.

### Effect of Cellular Stress on L1 Promoter Activity

**Figure 2:**
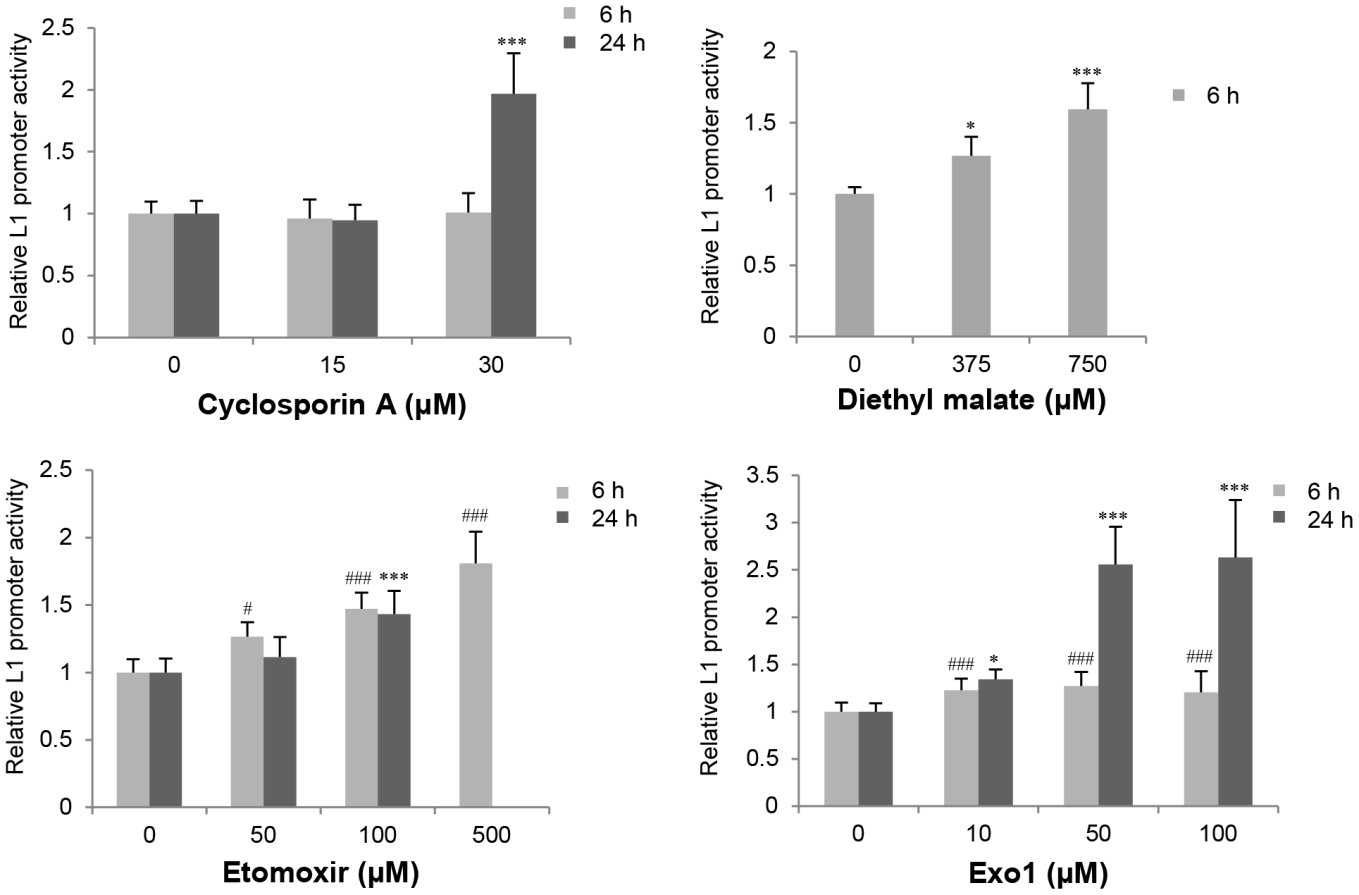
Effects of cellular stress induced by chemicals on L1 promoter activity in HepG2 cells. L1 promoter activity 6 and 24 hours after treatment was evaluated by the luciferase reporter gene assay. Data are represented as the average ratio (± SD) of the L1 promoter activity in the presence of the test compound relative to that in the presence of the vehicle control from at least three independent experiments (in duplicate; n = 6 or more) in the case of cyclosporin A, two independent experiments (in duplicate; n = 4) in the case of etomoxir and diethyl malate, and at least four independent experiments (in duplicate; n = 8 or more) in the case of exo1. The experimental data using diethyl malate and 500 µM etomoxir are shown only after 6 hours because of overt cytotoxicity after a 24-hour exposure (data not shown). Statistically significant differences as compared with the vehicle control for 6-hour and 24-hour treatments are indicated by *and #, respectively (*or #, *p*<0.05; ***or ###, *p*<0.001).

Next, we evaluated 27 substances that, through various mechanisms of action, induce cellular stress, including oxidative stress, mitochondrial dysfunction, and endoplasmic reticulum (ER) stress (Table 2). Four cellular stress compounds (cyclosporin A, diethyl malate, etomoxir, and exo 1) elevated L1 promoter activity by >1.5-fold (*p*<0.05) after exposure for 6 and/or 24 hours (Fig. 2).

**Table 2:** Effects of cellular stress induced by chemicals on L1 promoter activity.

Mechanism of action		Compound name	L1 promoter activity	
			6 h	24 h
Oxidative stress	Reactive oxygen species production	Carbon tetrachloride	N.S.	N.S.
		2,3-Dimethoxy-1,4-naphthoquinone	N.S.	N.D.
		Iodoacetamide	+	N.S.
		Menadione	N.S.	N.D.
		1-Methyl-4-phenyl-1,2,3,6-tetrahydropyridine	N.S.	N.S.
	Glutathione depletion	Diethyl malate	+ +	N.D.
		Phorone	+	N.S.
Mitochondrial dysfunction	Respiratory chain inhibitor	Antimycin A	N.S.	N.S.
		3-Nitropropionic acid	N.S.	N.S.
		Oligomycin	N.S.	−
		Rotenone	N.S.	N.S.
		Thenoyltrifluoroacetone	+	N.S.
	Mitochondrial permeability transition inducer	Citrinin	N.S.	+
	Mitochondrial membrane permeabilization	Cyclosporin A	N.S.	+ +
		Lonidamine	N.S.	N.S.
	β -oxidation inhibitor	1-Cyclopropanedicarboxylic acid	N.S.	− −
		Etomoxir	+ +	+
		4-Pentenoic acid	N.S.	− −
		Valproic acid	N.S.	− −
Endoplasmic reticulum stress	Calcium ionophore	A23187	− −	− −
		Ionomycin	− −	− −
		Tunicamycin	− −	−
	ER-Golgi transport inhibitor	Brefeldin	−	N.D.
		Exo1	+	+ +
Cell proliferation	Growth factor	EGF	N.S.	−
Steroidogenesis activator	Aldosterone secretion stimulator	Angiotensin II	N.S.	N.S.
	cAMP inducer	Forskolin	N.S.	− −

The effects of treatment for 6 and 24 hours on L1 promoter activity relative to the vehicle control were evaluated by luciferase reporter gene assay. For symbol definitions see Table 1.

Among the cellular stresses, oxidative stress is induced by diethyl malate, iodoacetamide, and phorone, all of which increased L1 promoter activity 1.2- to 2-fold. Cyclosporin A, which induced L1 promoter activity at 24 hours, inhibits mitochondrial membrane permeabilization [43] and is a drug frequently used in organ transplants as an immunosuppressant. Etomoxir, which enhanced L1 promoter activity by >1.5-fold after 6 hours of treatment at a concentration of 500 µM, induces mitochondrial dysfunction by inhibiting β-oxidation [44]. Exo1, which increased L1 promoter activity at 24 hours (Fig. 2), is an ER-Golgi transport inhibitor [45].

### Effect of Drugs on L1 Promoter Activity

**Figure 3:**
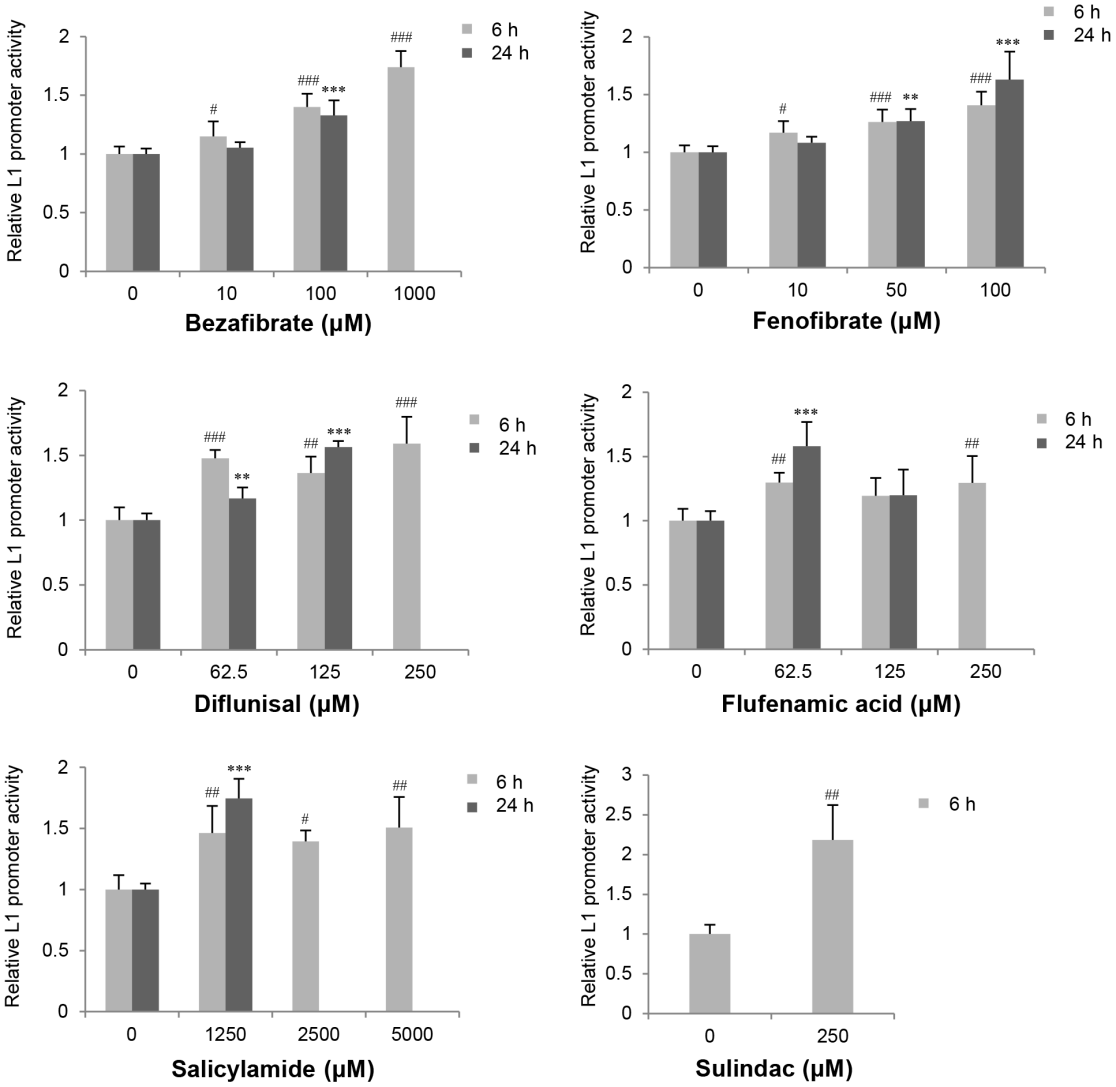
Effects of commercially available drugs on L1 promoter activity in HepG2 cells. L1 promoter activity 6 and 24 hours after treatment was evaluated by the luciferase reporter gene assay. Data are represented as the average ratio (± SD) of the L1 promoter activity in the presence of the test compound relative to that in the presence of the vehicle control from two independent experiments (in quadruplicate; n = 8) for bezafibrate and fenofibrate and from two independent experiments (in duplicate; n = 4) for diflunisal, flufenamic acid, salicylamide, and sulindac. The experimental data using sulindac are shown only after 6 hours because overt cytotoxicity occurred after a 24-hour exposure (data not shown). Statistically significant differences as compared with the vehicle control for 6-hour and 24-hour treatments are indicated by *or #, respectively (#, *p*<0.05; **or ##, *p*<0.01; ***or ###, *p*<0.001).

We used the L1 reporter gene assay to evaluate 47 drugs, 44 of which are now commercially available. These drugs have various pharmacological effects such as antilipemic, anticholesteremic, antifungal, antipyretic analgesic, antihistamine, antihypertensive, and diuretic effects (Table 3). Six drugs (bezafibrate, diflunisal, fenofibrate, flufenamic acid, salicylamide, and sulindac) increased L1 promoter activity >1.5-fold (*p*<0.05) after exposure for 6 and/or 24 hours (Fig. 3). Among 17 compounds which increased L1 promoter activity >1.2 fold (*p*<0.05) after exposure for 6 and/or 24 hours (Table 3), drugs with pharmacological effects such as peroxisome proliferator-activated receptor α- (PPARα) agonists (bezafibrate, clofibrate and fenofibrate) and non-steroidal anti-inflammatory drugs (NSAIDs; diflunisal, flufenamic acid, salicylamide, and sulindac) were especially likely to induce L1 promoter activity.

**Table 3:** Effects of drugs on L1 promoter activity.

Pharmacology		Compound name	L1 promoter activity	
			6 h	24 h
Antilipemic	PPARα agonist	Bezafibrate	+ +	+
		Clofibrate	N.S.	+
		Fenofibrate	+	+ +
		WY-14643^a^	N.S.	−
Anticholesteremic	HMG-CoA reductase inhibitor	Fluvastatin	N.S.	+
		Mevastatin^a^	N.S.	N.S.
		Pravastatin	+	N.S.
		Simvastatin	N.S.	N.S.
Anti-steroid drug (adrenocortical carcinoma)	Steroidogenesis inhibitor	Aminoglutethimide	N.S.	+
		o.p’- Dichlorodiphenyldichloroethane	−	N.S.
		Metyrapone	+	+
Hormone drug	Steroid hormone	Progesterone	−	−
	Sex hormone	17β-Estradiol	N.S.	N.S.
		Methyltestosterone	N.S.	−
	Thyroid hormone	Triiodothyronin	N.S.	−
Antifungal	Ergosterol synthesis inhibitor	Ketoconazol	−	− −
		Fluconazole	N.S.	N.S.
Antipyretic analgesic	COX inhibitor,nonsteroidal anti-inflammatory drug (NSAID)	Acetylsalicylic acid	+	− −
		Diclofenac	N.S.	− −
		Diflunisal	+ +	+ +
		Flufenamic acid	+	+ +
		Ibuprofen	N.S.	−
		Indometacin	−	− −
		Mefenamic acid	+	N.S.
		Salicylamide	+ +	+ +
		Sulindac	+ +	N.D.
		Zomepirac	N.S.	N.S.
Antipyretic analgesic		Acetaminophen	N.S.	+
Antihistamine	H1 histamine receptor agonist	(±)-Chlorpheniramine maleate salt	N.S.	N.S.
		Cyproheptadine hydrochloride sesquihydrate	− −	−
		Epinastine hydrochloride	− −	N.S.
		Ketotifen fumarate salt	−	N.S.
		Promethazine hydrochloride	−	N.D.
Antihistamine		Methapyrilene^a^	−	N.S.
Antitussive	Sigma receptor agonist	(*S*,*R*)-Noscapine	−	− −
Expectorant	Non-narcotic central antitussive	Guaiacol glyceryl ether	+	+
	Mucopolysaccharide synthesis inhibitor	Bromhexine hydrochloride	N.S.	N.S.
Hypertension	Ca^2+^ antagonist	Amlodipine besylate	N.S.	N.S.
		Nifedipine	N.S.	+
	Angiotensin II type 1 receptor blocker	Losartan	N.S.	N.S.
		Telmisartan	N.S.	N.S.
	Aldosterone antagonist	Spironolactone	N.S.	N.S.
Brain infarction	Anti-platelet agent	Clopidogrel	N.S.	N.S.
		Ticlopidine	N.S.	+
	Anticoagulant	Warfarin	N.S.	− −
Diuretics	Loop diuretic	Furosemide	N.S.	− −
	Thiazide diuretic	Trichlormethiazide	N.S.	− −

The effects of treatment for 6 and 24 hours on L1 promoter activity relative to the DMSO control were evaluated by luciferase reporter gene assay.

a not commercially available. For symbol definitions see Table 1.

### Comparison of the Effects of Compounds on L1 Promoter Activity between HepG2 Cells and HeLa Cells

**Figure 4:**
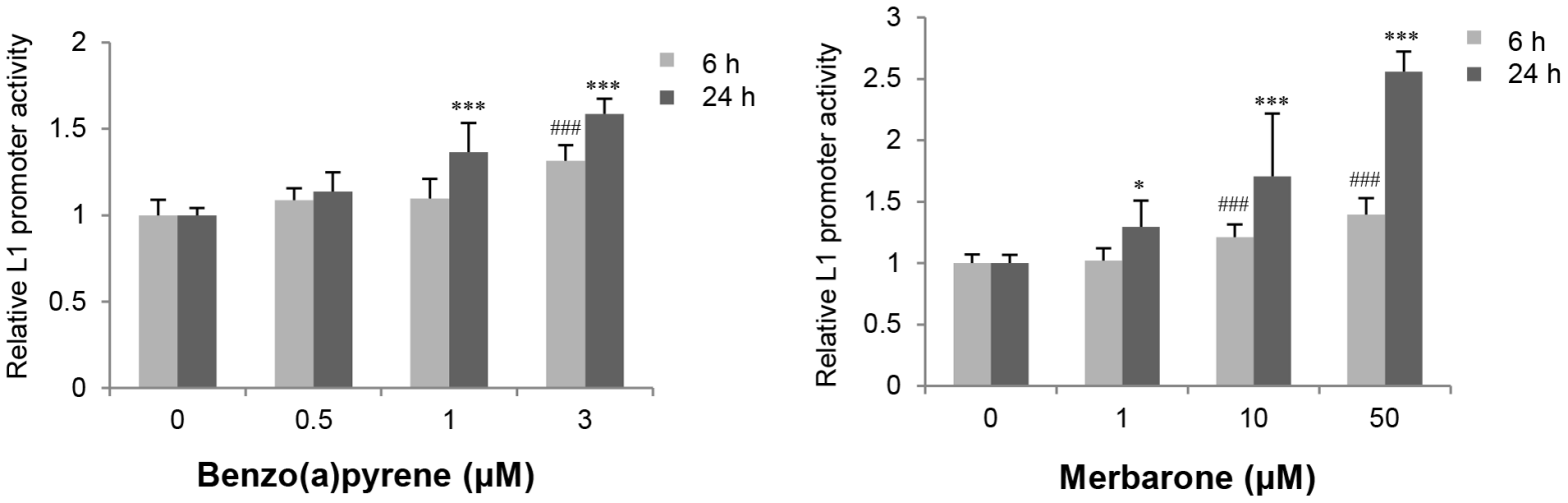
Effects of benzo[a]pyrene and merbarone on L1 promoter activity in HeLa cells. L1 promoter activity 6 and 24 hours after treatment was evaluated by the luciferase reporter gene assay. Data are represented as the average ratio (± SD) of the L1 promoter activity in the presence of the test compound relative to that in the presence of the vehicle control from two independent experiments (in quadruplicate; n = 8). Statistically significant differences as compared with the vehicle control for 6-hour and 24-hour treatments are indicated by *and #, respectively (*p<0.05; ***or ###, *p*<0.001).

To examine whether the chemical-enhanced L1 promoter activity is observed in HeLa cells, we performed the same experiments using HeLa cells (Fig. S2). Two test compounds, benzo[a]pyrene and merbarone, each of which elevated L1 promoter activity in HepG2 cells, were chosen for this experiment. These two compounds also enhanced the L1 promoter activity in HeLa cells in a dose-dependent manner (Fig. 4). All the data for the analysis of these two compounds in HeLa cells are shown in Dataset S2.

### A Novel Dual Secreted Luciferase L1 Retrotransposition Assay

To examine whether the enhancement of L1 promoter activity might be correlated with mobilization of the L1, we conducted a cell culture retrotransposition assay. We designed a novel high-throughput assay using a secreted Gaussia luciferase retrotransposition cassette to determine retrotransposition in human cells. Gaussia luciferase (GLuc), isolated from the marine copepod *Gaussia princeps*, catalyzes the oxidation of the substrate coelenterazine, the same substrate used by Renilla luciferase [37]. As a retrotransposition reporter, Gaussia has significant advantages over other luciferases. Codon-optimized GLuc generates over 1000-fold more signal intensity than Firefly and Renilla luciferases, and is very stable in culture medium with a half-life of about 6 days [46]. Also, since most L1 insertions are severely 5′ truncated, the small size of GLuc (558 bp, compared with 936 bp for RLuc and 1653 bp for FLuc) means fewer events go undetected due to loss of reporter cassette sequence at the time of insertion. Importantly, over 90 percent of the protein is secreted so retrotransposition events can be detected by simply sampling the media without disruption of the cells. This allows for the assessment of retrotransposition at multiple time points and the ability to carry out additional experiments, such as measuring cell viability, on the same population of cells. Thus, many compounds can be screened simultaneously under a variety of concentrations in a 96-well format.

**Figure 5:**
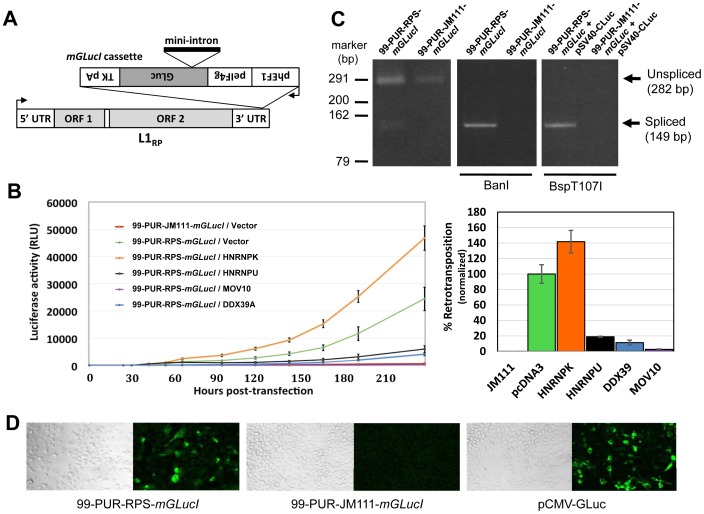
A novel secreted Gaussia luciferase L1 retrotransposition assay system. (A) A novel Gaussia luciferase cassette in a human L1. The *mGLucI* retrotransposition cassette was cloned into the 3′ UTR of L1_RP_ in the antisense orientation. The cassette contains the GLuc gene interrupted by an antisense mini-intron with an EF-1α/eIF4g hybrid promoter (phEF1/peIF4g) and the TK poly(A) signal (TK pA). GLuc can be expressed only when retrotransposition occurs. (B) Coexpression in HEK293T cells of selected cDNA constructs, previously determined to alter retrotransposition levels in the EGFP reporter assay (right panel; [40]), similarly affect retrotransposition in the GLuc assay (left panel). Fifty µl of media were sampled from each well (four replicate wells for each construct) at the indicated hourly time points over the course of 10 days and tested at the conclusion of the experiment (RLU: Relative Light Units). EGFP assays were performed at approximately 115 hours and results were normalized to cotransfected 99-PUR-RPS-EGFP reporter construct and empty vector (pcDNA3). Data for the earliest time-points sampled are reproduced in Fig. S3. (C) Retrotransposition in HeLa cells is confirmed by PCR using primers that flank the intron of the GLuc reporter gene. The presence of a band of 149 bp is diagnostic for intron removal. A 283-bp band is amplified from the transfected 99-PUR-RPS-*mGLucI* or 99-PUR-JM111-*mGLucI* plasmid. (D) Immunofluorescence analysis of transfected and fixed HEK293T cells showing obvious expression of GLuc from retrotransposition events formed by 99-PUR-RPS-*mGLucI* but not 99-PUR-JM111*-mGLucI*. Constitutive luciferase expression from pCMV-GLuc is also shown (right), detected by α-Gaussia antibody.

The secreted GLuc retrotransposition cassette consists of the GLuc gene interrupted by an antisense mini-intron, the EF-1α/eIF4g hybrid promoter (which is stronger than the CMV promoter), and the TK poly(A) signal (Fig. 5A). The GLuc retrotransposition cassette was cloned into the 3′ UTR of L1_RP_ in the opposite orientation. L1_RP_ is one of the most active human L1s [36] and belongs to the young Transcribed-Active (Ta) subfamily [47]. We cloned L1_RP_ containing the GLuc retrotransposition cassette into an episomally replicating mammalian expression vector that expresses a puromycin resistance gene. The resultant plasmid was named 99-PUR-RPS*-mGLucI*.

All evidence indicates that Gaussia luciferase secreted into the media serves as an effective read-out of accumulated retrotransposition events. GLuc is expressed only when the intron is spliced from the chimeric L1-reporter RNA, the RNA is reverse transcribed, and its cDNA is inserted in the genome by retrotransposition. Testing initially for efficacy in 6-well plates, we cotransfected 99-PUR-RPS*-mGLucI* with empty vector or cDNA constructs whose protein products were previously determined to alter EGFP-reporter retrotransposition in HEK293T cell culture (Fig. 5B; [38], [40]). At 24 hours post-transfection 3 mls of media was replaced in each well, and sampling was begun. Sampling 50 µl of media from 4 replicate wells for 10 days, a strong and steady accumulation of luminescent signal was detected for 99-PUR-RPS*-mGLucI* but not for 99-PUR-JM111*-mGLucI*, a mutant L1 construct defective for retrotransposition [8]. Coexpression of 99-PUR-RPS*-mGLucI* with cDNAs of MOV10, DDX39, and HNRNPU reduced luminescence levels compared with empty vector alone in a manner comparable to that previously determined by the EGFP-reporter assay (performed at 5 days post-transfection). Heterogeneous nuclear ribonucleoprotein K (hnRNPK) increased retrotransposition as previously reported [40].

Kroutter et al. noted that earliest L1 retrotransposition events are detected only at 32 hours post-transfection [48]. By treating HeLa cells with reverse transcriptase inhibitor d4t, the authors were able to inhibit retrotransposition at different time points and perform a time-course analysis using the neomycin reporter assay [8]. Supporting this finding, we observed by microscopy no retrotransposition-positive HEK293T cells prior to about 30 hours with the EGFP retrotransposition assay. Similarly, following replacement of the media at 24 hours post-transfection, detectable amounts of secreted Gaussia luciferase began to accumulate in the media after 30 hours and the commencement of sampling (Fig. S3).

To confirm that retrotransposition occurs in our system, we amplified by PCR genomic DNA isolated from transfected HeLa cells using primers that flank the mini-intron of *mGLucI* (Fig. 5C). An intronless product of 149 bp was seen in samples transfected with 99-PUR-RPS*-mGLucI* but not with its ORF1 mutant. By digesting isolated DNA with restriction enzymes that cut within the mini-intron prior to PCR, we were able to select against the 282-bp unspliced band amplified from plasmid DNA and increase signal from the spliced product.

While most Gaussia luciferase is secreted into the media some remains in the cells. To detect this residual luciferase, three days post-transfection we fixed cells with paraformaldehyde and performed immunofluorescence with an antibody against Gaussia luciferase (Fig. 5D). Almost no signal was detected from HEK293T cells transfected with the mutant construct 99-PUR-JM111*-mGLucI*, while fluorescent-positive cells transfected with 99-PUR-RPS*-mGLucI* were numerous.

### High-throughput Testing of Compounds for Effects on Retrotransposition

**Figure 6:**
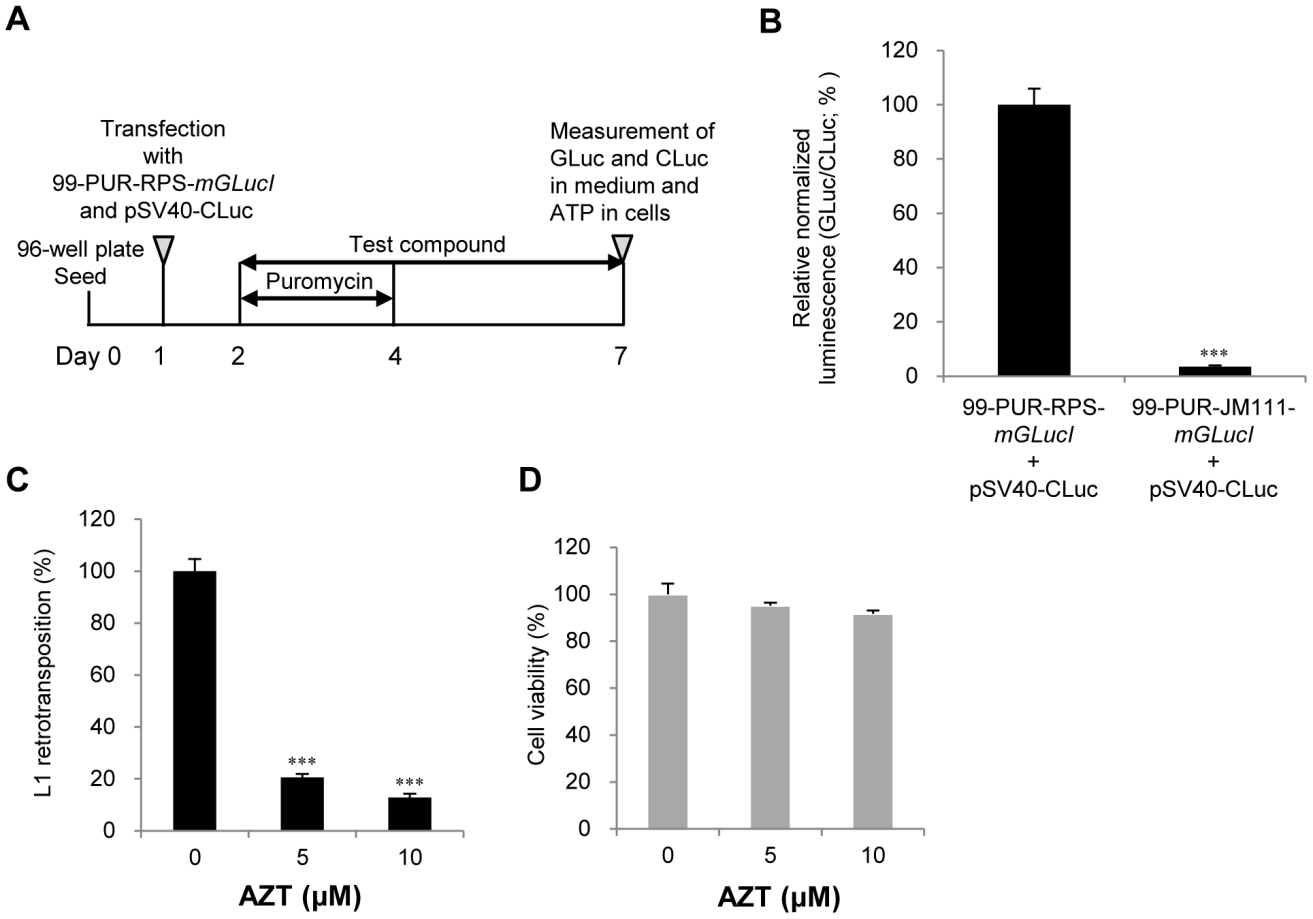
The dual secreted luciferase L1 retrotransposition assay. (A) Timeline of dual secreted luciferase L1 retrotransposition assay. HeLa cells transfected with 99-PUR-RPS-*mGLucI* and pSV40-CLuc were exposed to a test compound and puromycin for 2 days. The cells were then exposed to the test compound alone for an additional 3 days. GLuc and CLuc luminescence in the medium and cellular ATP were measured with a luminometer. GLuc luminescence indicates L1 retrotransposition activity. CLuc luminescence was used for normalization as an internal control. Cell viability was evaluated by cellular ATP content. (B) Effect of JM111 (L1_RP_ ORF1 mutant) on L1 retrotransposition activity. Either 99-PUR-RPS-*mGLucI* or 99-PUR -JM111-*mGLucI* containing two missense mutations in L1_RP_ ORF1 and pSV40-CLuc were co-transfected. L1 retrotransposition activity is represented as normalized luminescence. ***p<0.001. (C, D) Effect of the reverse transcriptase inhibitor azidothymidine (AZT) on L1 retrotransposition activity (C) and cell viability (D). L1 retrotransposition activity and cell viability are indicated as the percentage of the vehicle control. Data in (B–D) are the mean (± SD) from four independent experiments and are expressed relative to each control. ***p<0.001.

The timeline for the retrotransposition assay used to query the effects of chemical compound exposure is illustrated in Figure 6A. HeLa cells were seeded on a 96-well plate and cotransfected with 99-PUR-RPS-*mGLucI* and pSV40-CLuc, a construct that expresses Cypridina, a secreted luciferase isolated from the marine ostracod *Cypridina noctiluca* [39]. CLuc reacts with *Cypridina* luciferin, while GLuc reacts with coelenterazine; the two bioluminescences do not cross-react and are clearly distinguished in the sample media. Furthermore, both luciferases are considerably brighter than traditional Firefly luciferase reporters.

At 24 hours post-transfection, cells were exposed to puromycin and a test compound for 2 days. The cells were treated with the test compound alone for an additional 3 days. Finally, luminescence of both GLuc and CLuc in the medium and ATP content in the cells were measured. The effect of a compound on L1 retrotransposition activity was considered valid only when cell viability was >80% of the DMSO control value.

To validate the assay system for L1 retrotransposition in 96-well plates, 99-PUR-RPS-*mGLucI* and 99-PUR-JM111-*mGLucI* vectors were individually transfected along with pSV40-CLuc into HeLa cells. GLuc and CLuc were measured 6 days post-transfection. When the wild-type plasmid was transfected, strong GLuc luminescence was detected (Fig. S4). However, retrotransposition activity, i.e. GLuc signal, was abolished by the 99-PUR-JM111-*mGLucI* mutant, while CLuc signal remained strong (Fig. 6B, S4).

The reverse transcriptase inhibitor azidothymidine (AZT) inhibits reverse transcription by becoming integrated into the elongating cDNA sequence. AZT inhibits L1 retrotransposition as measured with a neomycin cassette [49]. We thus tested AZT in our dual secreted luciferase retrotransposition assay. AZT treatments significantly inhibited L1 retrotransposition; <20% of control activity was observed in the presence of either 5 or 10 µM AZT (Fig. 6C). Treatment with AZT did not, however, cause cytotoxicity (Fig. 6D). Together, these data show that this high-throughput dual secreted luciferase system for the detection of L1 retrotransposition is very useful for evaluating the effects of a variety of compounds on L1 retrotransposition in HeLa cells in a 96-well format.

### Analysis of the Dual Secreted Luciferase Assay for Screening the Effects of Compounds on L1 Retrotransposition

**Figure 7:**
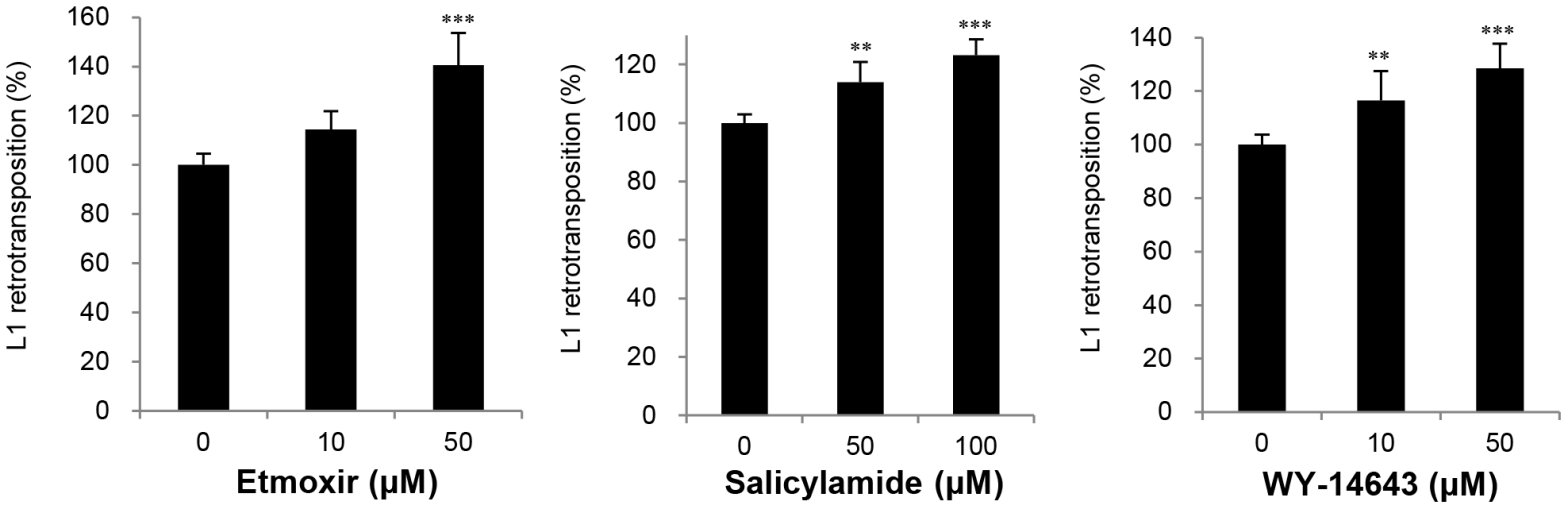
Effects of etomoxir, salicylamide, and WY-14643 on L1 retrotransposition. The effects of L1 retrotransposition activity after treatment for 5 days are shown. Data are the mean (± SD) from four independent experiments and are expressed relative to the vehicle control. **p<0.01; ***p<0.001.

We evaluated the effects of 26 compounds on L1 retrotransposition with the dual secreted luciferase assay. Twenty one of the test compounds were chosen from among the agents that enhanced L1 promoter activity in the L1 reporter gene assay. The remaining five compounds were randomly chosen from among those that did not enhance L1 promoter activity. Among the 21 compounds that elevated L1 promoter activity, etomoxir elevated L1 retrotransposition by 40.6% as compared with the control at 50 µM (*p*<0.0001; Fig. 7). Salicylamide, which is commonly used as an antipyretic analgesic, enhanced L1 retrotransposition activity by 23.1% at 100 µM (*p*<0.0001; Fig. 7). Although it did not elevate L1 promoter activity in the L1 reporter gene assay (Table 3), WY-14643, which is a PPARα agonist, increased L1 retrotransposition by 28.5% at 50 µM (*p*<0.05; Fig. 7). Bezafibrate, citrinin, colchicine, cyclosporin A, diflunisal, and exo1) significantly increased L1 retrotransposition activity to 110–120% of the control (*p*<0.0001; Dataset S3). All data for the dual secreted luciferase L1 retrotransposition assay as well as those for cell viability are shown in Dataset S3.

## Discussion

### Several Compounds, Including Commercially Available Drugs, Enhance L1 Transcription and Retrotransposition

We evaluated the effects of 95 compounds on L1 transcription using a L1 reporter gene assay. Of these compounds, 15 (8 of which are commercially available drugs) significantly increased L1 promoter activity by >1.5-fold at 6 and/or 24 hours. We then assessed the effects of compounds on L1 retrotransposition. The results revealed that 3 compounds significantly increased L1 retrotransposition activity by 20–40.6% (Fig. 7). The data described above were obtained by performing experiments using controls that were carefully designed for both transcription and retrotransposition. In both cases, to normalize the actual data, an internal control with luminescence was used to estimate the efficiency of transfection and to adjust for any possible effects of compound treatment on cytotoxicity or cell growth, and hence the number of cells in different wells. In addition, in all cases the reproducibility of the data was confirmed. Especially in the case of retrotransposition, we carefully designed and performed experiments at concentrations where cytotoxicity or altered cell growth was not observed. Although the resulting ratios of the increase in transcription and retrotransposition were not large, the statistically significant effects of these compounds on transcription and retrotransposition are meaningful.

### The Effects of these Compounds on Retrotransposition *in vivo* might be Larger than those Observed *in vitro*

Although our retrotransposition experiments were conducted *in vitro*, there is a possibility that the effects of these compounds on retrotransposition *in vivo* might be larger than those observed *in vitro*. Several factors suggest that this might be the case. First, benzo[a]pyrene, which is a DNA intercalator and an inducer of genotoxicity, induces both transcription [24] and retrotransposition [25] of L1. In our study, benzo[a]pyrene increased L1 transcription but did not increase L1 retrotransposition. We speculate that this difference may have been caused by the conditions of our retrotransposition assay. In particular, we evaluated retrotransposition in a short transient assay (i.e., 5 days), whereas the authors of the previous report used a stable cell line that could be reseeded and cultured for a long time (i.e., 14 days). Thus, we do not know what effects would be observed after long-term assessment.

A second factor that may lead to a difference in the effects seen *in vitro* versus those *in vivo* has to do with the L1. Here we used L1_RP_, which has very high retrotransposition activity [36]. Because of this high basal retrotransposition activity of L1_RP_, we may not have been able to detect enhanced retrotransposition activity, even if it was substantial. Thus, compounds that only slightly elevated L1 retrotransposition might induce retrotransposition more *in vivo* than would have been expected based on experiments *in vitro*. Moreover, it is estimated that 80 to 100 L1s remain potentially active for retrotransposition in the average diploid human genome [2], [3]: exposure to a compound could activate some or all of these elements, and each to an unknown degree.

The third factor concerns the difference in conditions between *in vivo* and *in vitro* assays. In the case of the PPARα agonists, bezafibrate and fenofibrate enhanced L1 transcription and bezafibrate and WY-14643 slightly increased L1 retrotransposition. Long-term exposure of mice to WY-14643 decreases the DNA methylation in the liver of LINE1s, LINE2s, and intracisternal A particles (IAPs); the latter is an active retrotransposon in mice [50]. Furthermore, DNA methylation inhibits L1 expression, and DNA hypomethylation increases L1 retrotransposition activity [51]–[53]. Accordingly, PPARα agonists may activate L1 retrotransposition in addition to L1 transcription by decreasing DNA methylation. The retrotransposition assay we established in this study is a transient one that uses an episomally replicating mammalian expression vector, so this assay may underestimate the effect of these compounds on DNA methylation *in vivo*. A stable assay system that allows us to assess retrotransposition *in vivo*, including effects that result from epigenetics, will be useful to evaluate the effects of compounds on L1 retrotransposition.

### The Novel Dual Secreted Luciferase L1 Retrotransposition Assay is Useful for High-throughput Screening

Cell culture assays for retrotransposition are the most important tools for studying LINE1 elements in humans and mice. These assays report the final step of retrotransposition, the number of cells in a total population of cells that receive an insertion event. The first assay to measure retrotransposition in cells was developed over 25 years ago [54]. Transposition of a yeast Ty1 retrotransposon from a donor plasmid to a mutant HIS3 gene in a target plasmid resulted in a His+ revertant phenotype. Spliced removal of an artificial intron engineered into the Ty1 element confirmed movement had occurred through an RNA intermediate. Subsequently, Heidmann et al. developed the *neoRT* reporter cassette, containing a neomycin phosphotransferase (*neo*) gene and an artificial intron placed between the promoter and *neo* coding sequence [55]. Retrotransposition events were detected by formation of neomycin (G418)-resistant colonies in cell culture.

Freeman et al. redesigned *neoRT* [56]. Their cassette (*mneoI*) included *neo* interrupted by antisense γ-globin intron 2. Moran et al. inserted *mneoI* in opposite transcriptional orientation into the 3′ untranslated regions (UTRs) of L1.2 and LRE2 [8], elements previously identified as likely precursors of germ line L1 insertions into patient factor VIII [5] and dystrophin genes [16], respectively. High levels of retrotransposition were detected in tumor cell culture. In subsequent years, assay sensitivity was further improved by the discovery of more active precursor L1s: L1.2 and LRE2 were followed by incrementally more active L1.3 [41], L1_RP_ [36], LRE3 [57], and LRE4 [58]. Codon and sequence optimization of synthetic human and mouse L1s also boosted retrotransposition potential [59]–[62]. Rangwala et al. details the history of cell culture retrotransposition assays [63].

Limitations exist for each of the L1 cell culture assays in widespread use. The *mneoI* assay requires 3 weeks for antibiotic selection. A blasticidin S-deaminase gene (*bsr*) reporter cassette cloned in L1_RP_ and a vector that constitutively expresses puromycin cut antibiotic selection time in half [34]. Nevertheless, both assays remain labor-intensive, cannot be used with poorly adherent cell lines, and are prone to overestimates of retrotransposition due to satellite colonies reseeded from retrotransposition-positive parent colonies during the prolonged period of antibiotic selection. Ostertag et al. reduced assay time to 5 days by replacing the antibiotic resistant cassette with one expressing EGFP [38]. However, this assay requires access to a flow cytometer. Recently Xie et al. developed a “third-generation” dual-luciferase assay using the FLuc gene as reporter for L1 retrotransposition and RLuc gene for transfection normalization [64]. The approach is amenable to high-throughput assays, and data are obtainable at several days by lysing cells.

None of the current assays permit easy study of retrotransposition kinetics and the performance of multiple assays in a single well. Different wells may be sampled sequentially to establish a time-course series [38]. More effectively, Kroutter et al. employed a reverse-transcriptase inhibitor to halt retrotransposition at selected time points in a single experiment, permitting cells for all time-points to be harvested together at the conclusion of the experiment [48].

Cognizant of assay limitations, we designed a “fourth generation” cell culture retrotransposition assay that is easy to execute, sensitive, amenable to high-throughput analyses, and permissive for time-course sampling from a single transfected well. We constructed a novel retrotransposition cassette reporter (*mGLucI*) containing a modified Gaussia luciferase gene. The JM111 mutant, which exhibits almost no retrotransposition activity because of the introduction of two missense mutations in ORF1 [8], [38], [64], and AZT, which inhibits L1 retrotransposition [49], dramatically reduced the L1 retrotransposition activity, supporting the validity of the system. This assay system is very useful for screening many compounds at a variety of concentrations without disruption of the cells. Here we evaluated the effects of compounds on both L1 retrotransposition and cell viability using the same sample.

### Genotoxic Agents and Cellular Stress Induced L1 Transcription and/or Retrotransposition as Previously Described

DNA damage induced by genotoxic stresses such as UV light [24], X-rays [27], gamma radiation [26], and some chemicals [24], [65] activates L1 transcription and/or retrotransposition. In our study, some genotoxic agents increased L1 promoter activity (Fig. 1). Among the tested genotoxic agents, only colchicine, which inhibits microtubule assembly, increased L1 retrotransposition slightly, albeit significantly.

There are two possible reasons why these genotoxic agents enhance L1 retrotransposition. First, they may enhance retrotransposition through induction of DNA breaks, which would allow the integration of L1 without involving its endonuclease [66], [67]. Second, genotoxic agents might induce the expression of certain transcription factors, the binding sites for which are contained in the L1 5′ UTR. Accordingly, some transcription factors, such as SRY family members [68], YY1 [69], RUNX3 [70], and p53 [71], might activate L1 transcription through their binding to the L1 5′ UTR. Indeed, it is known that genotoxic agents activate p53 protein [72], [73].

Cellular stress has some effect on L1 retrotransposition, as exemplified by the increase in L1 retrotransposition that results from oxidative stress induced by H_2_O_2_ [28]. L1 ORF1 protein is localized in cytoplasmic stress granules that are formed under stress conditions [34], suggesting a possible physical connection between retrotransposition and stress. In our study, the compounds that induce mitochondrial dysfunction (such as cyclosporin A, etomoxir, and citrinin) and those that induce ER stress (such as exo1) also induced L1 retrotransposition, whereas oxidative stress-inducing chemicals only increased L1 promoter activity (Table 1).

### Risk Assessment of Enhanced Retrotransposition Induced by Compound Exposure should be Considered During Drug Discovery

During drug development, compounds are selected based on the assessment of their therapeutic activity and toxicity characteristics as well as pharmacokinetics (namely, absorption, distribution, metabolism, and excretion). However, the risk of retrotransposition has not yet been considered as part of drug safety assessment, even though there are several reports that chemical-induced stress activates L1 transcription and retrotransposition and that the integration of retrotransposition into genes causes disease (reviewed in [12], [14], [74]). In particular, salicylamide is widely used as an antipyretic analgesic, for which the peak blood concentration is 24.8 µM [75]. We observed the enhancement of retrotransposition by this drug at 50–100 µM *in vitro*. Although this range is higher than the peak blood concentration for salicylamide, this drug has the potential to induce retrotransposition *in vivo* because of long-term exposure from frequent doses. Accordingly, we believe that the data presented here provide a new paradigm for drug assessment.

Because this L1 retrotransposition assay is carried out *in vitro*, we should confirm these effects on L1 retrotransposition *in vivo*. L1 retrotransposition can be induced in skin tumors of L1 transgenic mice after exposure to 7,12-dimethylbenz[a]anthracene and 12-O-tetradecanoylphorbol-13-acetate [76]. We expect that a new *in vivo* assay system will be able to evaluate the enhancement of retrotransposition by compounds not only in tumors but also in germ line and somatic cells.

In conclusion, our study demonstrated that several chemicals and drugs increased L1 transcription and retrotransposition significantly and that certain chemical- and drug- induced stresses might have the potential to cause genomic mutations by inducing L1 mobilization. Because either active or passive exposure to drugs may activate retrotransposons *in vivo*, we propose that new assessment protocols should be undertaken during drug safety evaluation and environmental risk assessments of chemicals.

## Supporting Information

Figure S1 **Firefly luciferase (FLuc) and Renilla luciferase (RLuc) expression in the L1 reporter gene assay in HepG2 cells.** Relative FLuc luminescence, relative RLuc luminescence, and relative normalized luciferase luminescence at 6 hours (A) and 24 hours (B) after treatment with vehicle control (DMSO) in the L1 reporter gene assay using HepG2 cells. FLuc and RLuc indicate L1 promoter activity and expression of the internal control, respectively. Data are the mean ± SD from four independent experiments. TK pro-R: pGL4.74; no pro-F: pGL4.11; L1 5′ UTR-F: pGL4.11-L1.3 5′ UTR.
(TIF)

Figure S2 **FLuc and RLuc expression in L1 reporter gene assay in HeLa cells.** Relative FLuc luminescence, relative RLuc luminescence, and relative normalized luciferase luminescence at 6 hours (A) and 24 hours (B) after treatment with the vehicle control (DMSO) in the L1 reporter gene assay using HeLa cells. FLuc and RLuc indicate L1 promoter activity and an internal control, respectively. Data are the mean ± SD from four independent experiments. TK pro-R: pGL4.74; no pro-F: pGL4.11; L1 5′ UTR-F: pGL4.11-L1.3 5′ UTR.
(TIF)

Figure S3 **Data for the earliest sampling time-points of the time-course GLuc retrotransposition assay shown in** **Fig. 5B** **.** See legend for that figure.
(TIF)

Figure S4 **GLuc and CLuc expression in the novel dual secreted luciferase L1 retrotransposition assay.** GLuc and CLuc luciferase luminescence in the novel L1 retrotransposition assay 6 days post-transfection. Either 99-PUR-RPS-*mGLucI* or 99-PUR -JM111-*mGLucI* containing two missense mutations in L1_RP_ ORF1 and pSV40-CLuc were co-transfected. GLuc can be expressed only when retrotransposition occurs. CLuc was used as an internal control. Data are the mean ± SD from six independent experiments. RLU: Relative Light Units.
(TIF)

Dataset S1 **Results of L1 reporter gene assay in HepG2 cells.** L1 promoter activity 6 and 24 hours after treatment was evaluated by luciferase reporter gene assay. L1 promoter activities are represented as a percentage relative to that of the vehicle control. Mean and SD from at least two independent experiments (in duplicate; n = 4 or more) are shown.
(XLS)

Dataset S2 **Results of L1 reporter gene assay in HeLa cells.** L1 promoter activity 6 and 24 hours after treatment was evaluated by the luciferase reporter gene assay. L1 promoter activities are represented as a percentage relative to that of the vehicle control. Mean and SD from two independent experiments (in quadruplicate; n = 8) are shown.
(XLS)

Dataset S3 **Results of L1 retrotransposition assay and cell viability.** L1 retrotransposition activity and cell viability after treatment are represented as a percentage relative to that of the vehicle control. Mean and SD from four independent experiments are shown.
(XLS)
