# Supplementary figures and images for: In Vitro Screening for Compounds That Enhance Human L1 Mobilization
Source: PLoS One. 2013 Sep 11;8(9):e74629. doi: 10.1371/journal.pone.0074629 (PMC3770661; doi:10.1371/journal.pone.0074629)

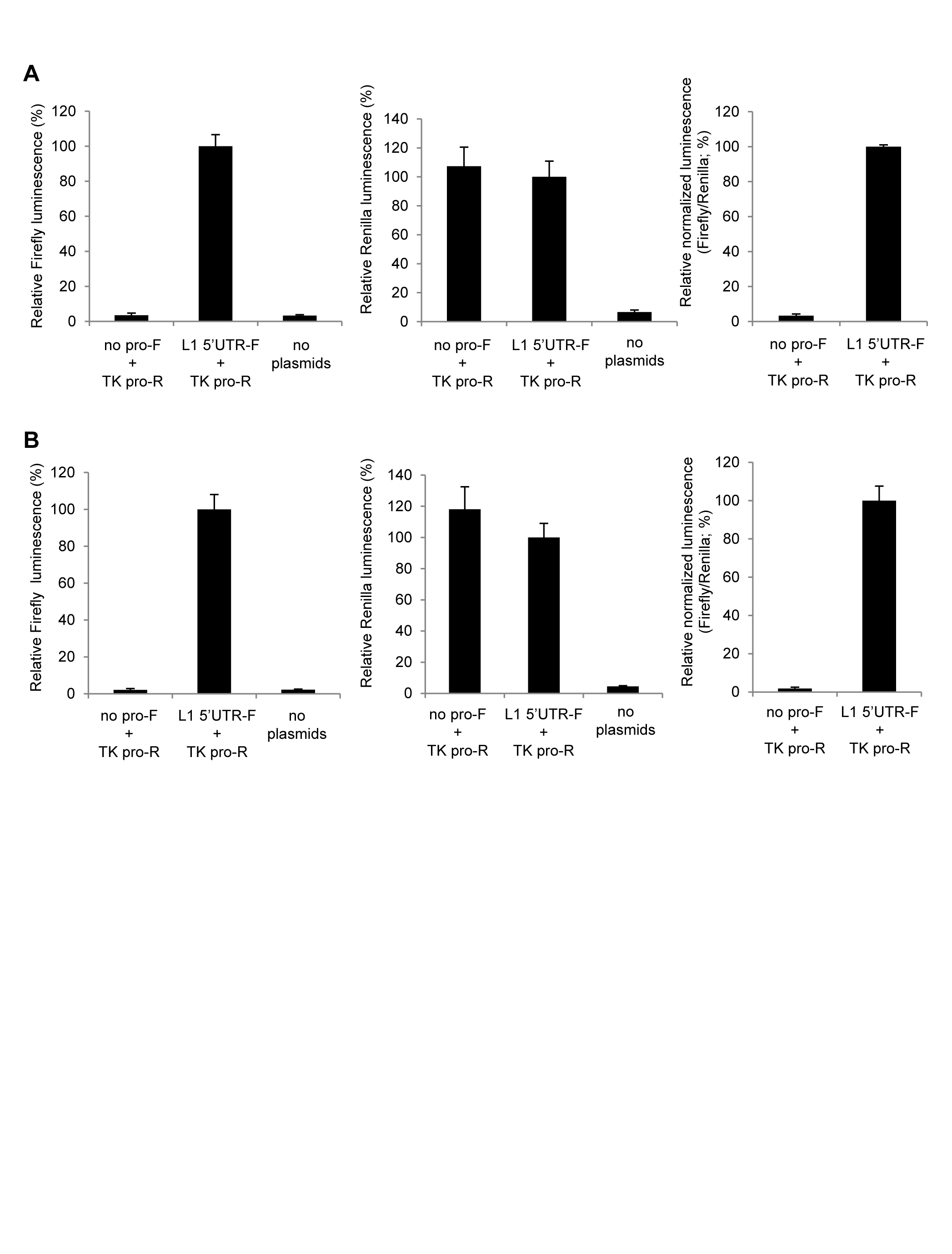

Supplement: Figure S1 — Firefly luciferase (FLuc) and Renilla luciferase (RLuc) expression in the L1 reporter gene assay in HepG2 cells. Relative FLuc luminescence, relative RLuc luminescence, and relative normalized luciferase luminescence at 6 hours (A) and 24 hours (B) after treatment with vehicle control (DMSO) in the L1 reporter gene assay using HepG2 cells. FLuc and RLuc indicate L1 promoter activity and expression of the internal control, respectively. Data are the mean ± SD from four independent experiments. TK pro-R: pGL4.74; no pro-F: pGL4.11; L1 5′ UTR-F: pGL4.11-L1.3 5′ UTR. (TIF) [file pone.0074629.s001.tif]

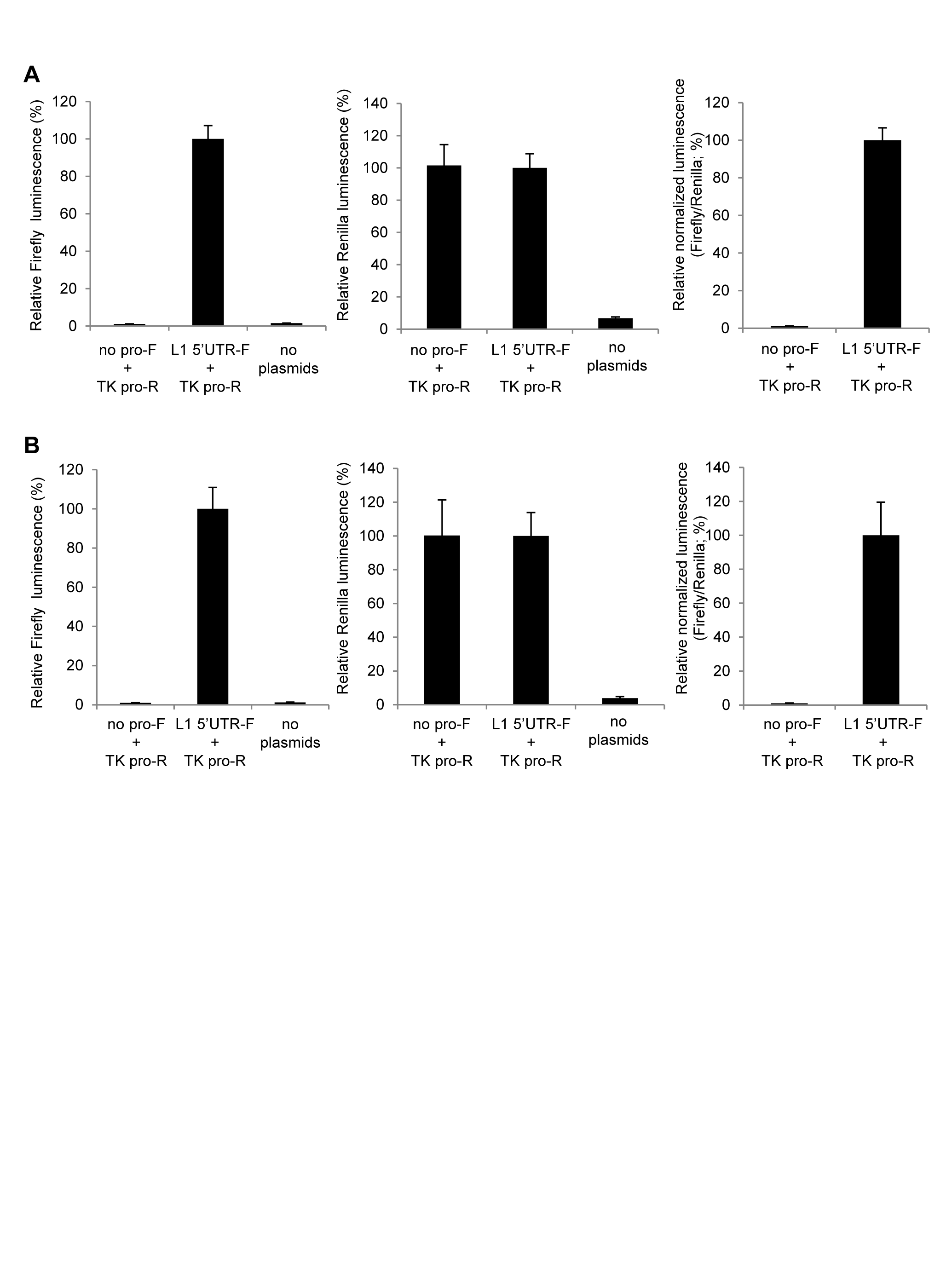

Supplement: Figure S2 — FLuc and RLuc expression in L1 reporter gene assay in HeLa cells. Relative FLuc luminescence, relative RLuc luminescence, and relative normalized luciferase luminescence at 6 hours (A) and 24 hours (B) after treatment with the vehicle control (DMSO) in the L1 reporter gene assay using HeLa cells. FLuc and RLuc indicate L1 promoter activity and an internal control, respectively. Data are the mean ± SD from four independent experiments. TK pro-R: pGL4.74; no pro-F: pGL4.11; L1 5′ UTR-F: pGL4.11-L1.3 5′ UTR. (TIF) [file pone.0074629.s002.tif]

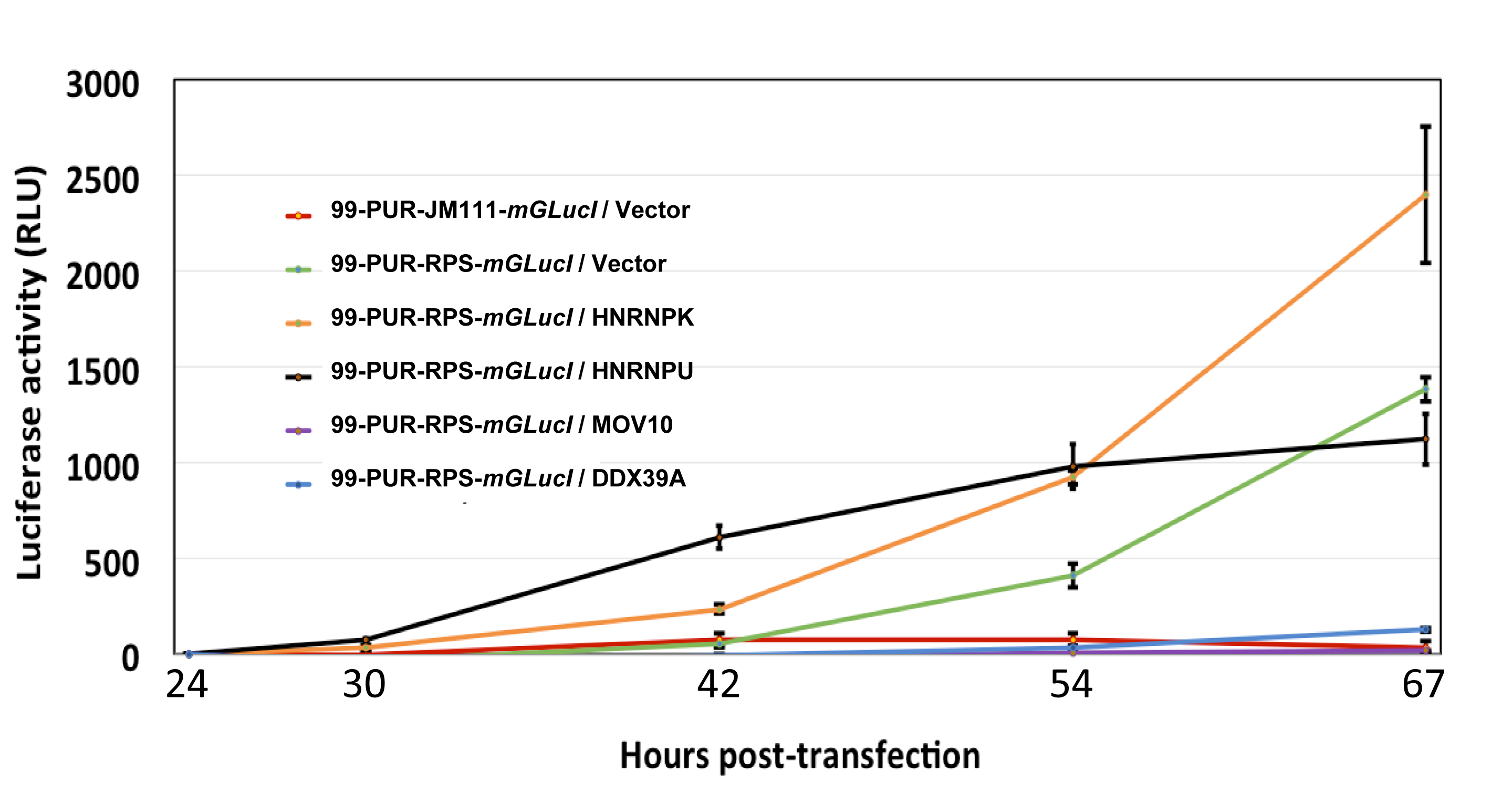

Supplement: Figure S3 — Data for the earliest sampling time-points of the time-course GLuc retrotransposition assay shown in Fig. 5B . See legend for that figure. (TIF) [file pone.0074629.s003.tif]

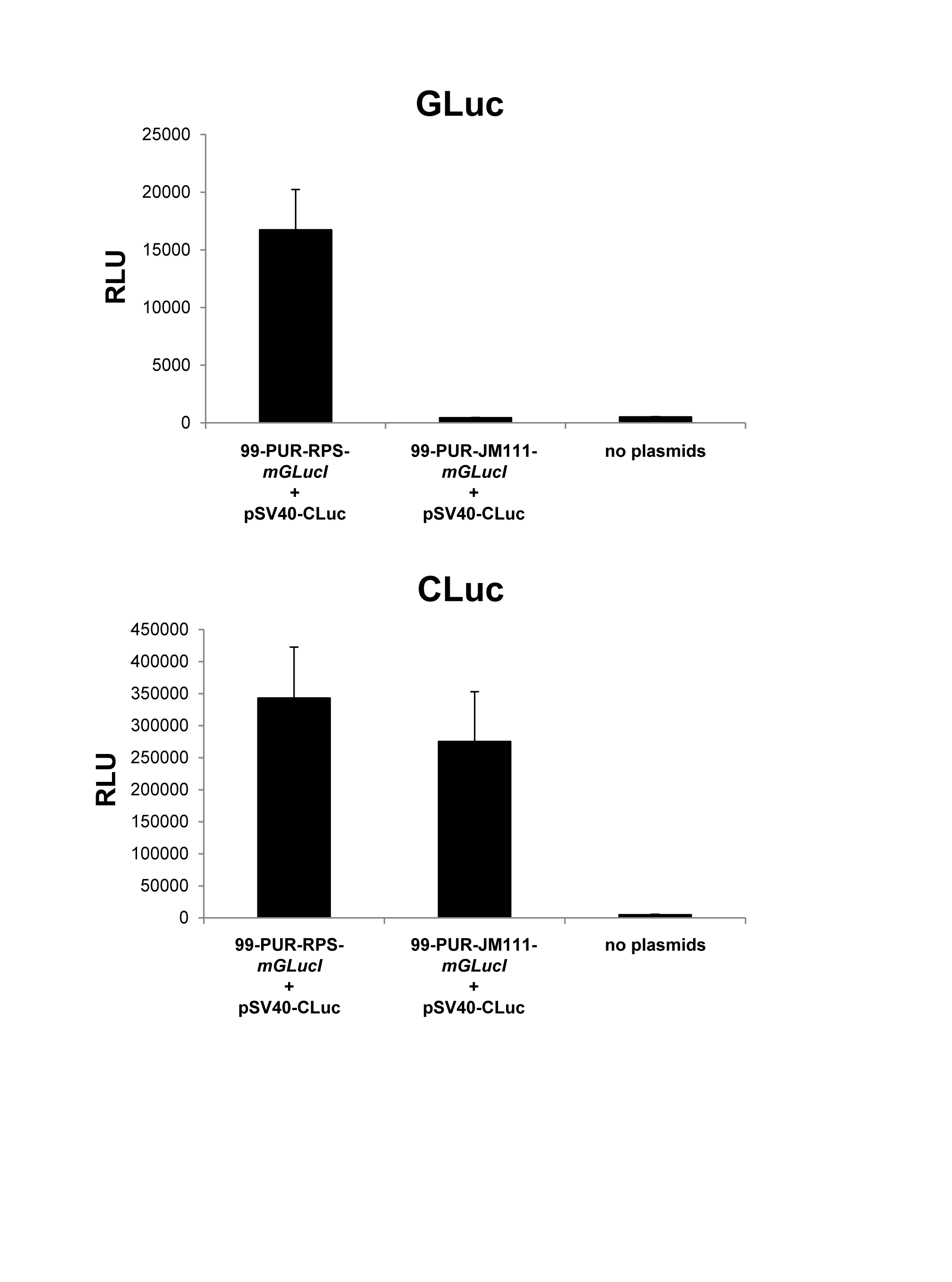

Supplement: Figure S4 — GLuc and CLuc expression in the novel dual secreted luciferase L1 retrotransposition assay. GLuc and CLuc luciferase luminescence in the novel L1 retrotransposition assay 6 days post-transfection. Either 99-PUR-RPS-mGLucI or 99-PUR -JM111-mGLucI containing two missense mutations in L1RP ORF1 and pSV40-CLuc were co-transfected. GLuc can be expressed only when retrotransposition occurs. CLuc was used as an internal control. Data are the mean ± SD from six independent experiments. RLU: Relative Light Units. (TIF) [file pone.0074629.s004.tif]
